# Changes in the Infrared Microspectroscopic Characteristics of DNA Caused by Cationic Elements, Different Base Richness and Single-Stranded Form

**DOI:** 10.1371/journal.pone.0043169

**Published:** 2012-08-24

**Authors:** Maria Luiza S. Mello, B. C. Vidal

**Affiliations:** Department of Structural and Physiological Biology, Institute of Biology, University of Campinas (Unicamp), Campinas, São Paulo, Brazil; University of Quebec at Trois-Rivieres, Canada

## Abstract

**Background:**

The infrared (IR) analysis of dried samples of DNA and DNA-polypeptide complexes is still scarce. Here we have studied the FT-IR profiles of these components to further the understanding of the FT-IR signatures of chromatin and cell nuclei.

**Methodology/Principal Findings:**

Calf thymus and salmon testis DNA, and complexes of histone H1, protamine, poly-L-lysine and poly-L-arginine (histone-mimic macromolecules) with DNA were analyzed in an IR microspectroscope equipped with an attenuated total reflection diamond objective and Grams software. Conditions including polypeptides bound to the DNA, DNA base composition, and single-stranded form were found to differently affect the vibrational characteristics of the chemical groups (especially, PO_2_^−^) in the nucleic acid. The antisymmetric stretching (ν_as_) of the DNA PO_2_^−^ was greater than the symmetric stretching (ν_s_) of these groups and increased in the polypeptide-DNA complexes. A shift of the ν_as_ of the DNA PO_2_^−^ to a lower frequency and an increased intensity of this vibration were induced especially by lysine-rich histones. Lysine richness additionally contributed to an increase in the vibrational stretching of the amide I group. Even in simple molecules such as inorganic phosphates, the vibrational characteristics of the phosphate anions were differently affected by different cations. As a result of the optimization of the DNA conformation by binding to arginine-rich polypeptides, enhancements of the vibrational characteristics in the FT-IR fingerprint could be detected. Although different profiles were obtained for the DNA with different base compositions, this situation was no longer verified in the polypeptide-DNA complexes and most likely in isolated chromatin or cell nuclei. However, the ν_as_ PO_2_^−^/ν_s_ PO_2_^−^ ratio could discriminate DNA with different base compositions and DNA in a single-stranded form.

**Conclusions/Significance:**

FT-IR spectral profiles are a valuable tool for establishing the vibrational characteristics of individualized chromatin components, such as DNA and DNA-polypeptide complexes in dried samples.

## Introduction

To discriminate between tissue and cell types under normal and pathological states, investment in research for the application of non-invasive label-free techniques such as vibrational microspectroscopy is growing rapidly [1]–[5]. In this context, Fourier Transform-Infrared (FT-IR) spectral microscopy is a useful tool. The designation FT-IR refers to the fact that a Fourier transform algorithm is required to convert the raw data into a spectral profile. After a beam of IR light passes through a sample, absorption profiles highlighting specific band peaks and troughs covering all of the wavenumbers are revealed at once. The FT-IR spectral profile is a molecular signature of a sample. Specific marker bands in different frequency regions in a FT-IR profile are associated with the vibration of particular chemical functional groups in a molecule.

Numerous comparisons of tumoral and healthy tissues or cell types under different physiological conditions have been reported by establishing the differences in the IR profiles (e.g., [6]–[18]). However, basic studies on the spectral FT-IR characteristics of the individualized chromatin components and their complexes in dried samples, such as those required for modern IR microspectroscopes, are still scarce. FT-IR spectroscopy, for example, has successfully diagnosed radiation damage in DNA and explained resistance in bacteria that survive very high doses of acute ionizing radiation [19].

In eukaryotic cells, chromatin is a complex architecture resulting from the association of DNA, histones, non-histone proteins, and RNA. The dynamic organization of the chromatin components regulates processes that are essential for normal cell physiology. Generally, the participation of DNA and RNA in FT-IR cell signatures is assumed from the vibrations of the nucleic acid phosphate groups and is particularly considered as one of the important biomarkers of cancer [6], [11]. According to Dukor [1], the changes in the nucleic acids as assessed by IR signatures are promoted by factors at the molecular level of these compounds, such as, the presence of extensive hydrogen bonding, an increase in symmetric phosphate stretching that indicates tighter nucleic acid packaging, and a decrease in the hydrogen bonding of associated C-OH protein groups. It is thus relevant to expect the influence of cations on the IR signatures of the phosphate anions even in inorganic phosphates used as models, and the influence of histone cationic groups - or at least of the reductionist models of lysine-rich and arginine-rich histones, such as poly-L-lysine (poly-L-K) and poly-L-arginine (poly-L-R), respectively, which bind electrostatically to the DNA phosphates - on the DNA FT-IR spectral profiles. Indeed, FT-IR studies have demonstrated that histone-mimicking cationic dendrimeres, which are potential transfection agents for gene delivery, strongly aggregate calf thymus DNA via major and minor grooves and the backbone phosphate groups of the nucleic acid [20]. Spectroscopic studies on ligand-phosphate binding in complexes between drugs (e.g. saffron components) and DNA have revealed an increase in intensity of phosphate-stretching vibrations and a certain degree of helix destabilization and drug intercalation [21].

Here, with the modern technology available for FT-IR microspectroscopy, we analyzed the influence of cations on the IR bands contributed by the phosphate group vibrations of sodium mono- and dibasic phosphates and potassium monobasic phosphate. We also analyzed the effect of poly-L-K, poly-L-R, histone H1 and protamine binding to DNA - and of a DNA single-stranded form - on the FT-IR spectral profiles of the DNA. Taking advantage of the present opportunity, other marker bands revealed in the FT-IR spectra in addition to the vibration of DNA phosphate groups, were analyzed when pertinent. The FT-IR spectra that were obtained and compared with the data previously reported in the literature (Tables 1,2) are intended to be a support for a library of the spectral profiles of chromatin components for further understanding and interpreting the differences in chromatin and whole cell nuclei under research conditions.

**Table 1:** Frequencies of the FT-IR absorption markers for DNA vibration groups as reported by different authors.

Assignment	Frequencies	Comment	Authors
	(wavenumber in cm^−1^)		
Guanine	1710	C = O stretching	[22]
	1716		[25]
Thymine	1700,1664		[22], [25]
	1663	C2 = O stretching	([28]-review)
	740	ν N = H	[18]
Adenine	1690, 1660–1640, 1610–1600, 1575		[22], [25]
	1610	C7 = N stretching	([20], [28]-review)
Cytosine	1492, ∼1300,1294		[25]
	1491	In-plane vibration	([20]-review)
Cytosine and Guanine	1527,1425,1374		[21]
	1527	In-plane vibration	([20]-review)
All bases	1400		[22]
DNA and RNA	1244	Phosphate antisymmetric stretching (ν_as_ PO_2_- phosphodiester groups)	[5], [18]
DNA	1225–1220	Main *B*-form marker; Phosphate antisymmetric stretching	([23]-review, [24], [25])
	1224	ν_as_ PO_2_- sensible to cationic polymer complexed to DNA	[24]
	1090–1085	Phosphate symmetric stretching; Insensitive to the *B*-to-*A* transition	([5], [13], [18], -reviews, [25])
	970–965	O-P-O bending	[13]
	970–950	*B*-form: singlet at 970	([23]-review)
	938	AT base pairs in *B*-form helices	([23]-review, [45])
Thymidine	1281	With S-type sugar conformation	[23]
	1275	With N-type sugars	[23]
Deoxyribose	1053	C-O deoxyribose stretching	([Bibr pone.0043169-Froehlich1] [20/28]-reviews)
	967	C-C and C-O of deoxyribose skeletal motions of DNA	[18]
	899–890	Deoxyribose ring vibration	([23]-review)
	834	Deoxyribose-phosphate, B-marker	([20]-review)
DNA sugar	842–820	Main S-type sugar marker	([23]-review, [29])
	780	Sugar-phosphate vibration	[18]

ν_as_, antisymmetric stretching; ν_s_, symmetric stretching.

**Table 2:** Frequencies of the FT-IR absorption markers for protein vibration groups as reported by different authors.

Assignment	Frequencies	Comment	Authors
	(wavenumber in cm^−1^)		
Mostly protein	3000–2800	ν CH region	[13]
	2958	ν_as_ CH_3_ and CH_2_	[18]
	2873	ν_s_ CH_3_	[18]
Protein	1659–1624	Amide I band heterogeneity groups in collagen triple helix (after peak fitting procedure)	[41]
	∼1656	Amide I	[18]
	1651	Amide I	[13]
	1640	Amide I-polyproline II dried film	[42]
	1629	Amide I – high H bonding; helical structure organization (collagen)	[41]
	1549	Amide II-stretching	[5]
		C-N (40%), bending N-H (50%), C-C (10%)	
	1547	Amide II	[13]
	∼1546	Amide II mode	[18]
	1449 and 1393	Symmetric and antisymmetric CH3 bending from amino acid side groups	[5]
	1400	ν COO^−^	[18]
	1313	Amide II band components	[18]
	∼1245	Amide III	[43]
	1155	ν C-O	[18]
Model - Nylon 6	∼1641, 1630	Ordered polyamide chain model	[41]

ν_as_, antisymmetric stretching; ν_s_, symmetric stretching.

## Results

### Inorganic Phosphates and Calf Thymus DNA

**Figure 1:**
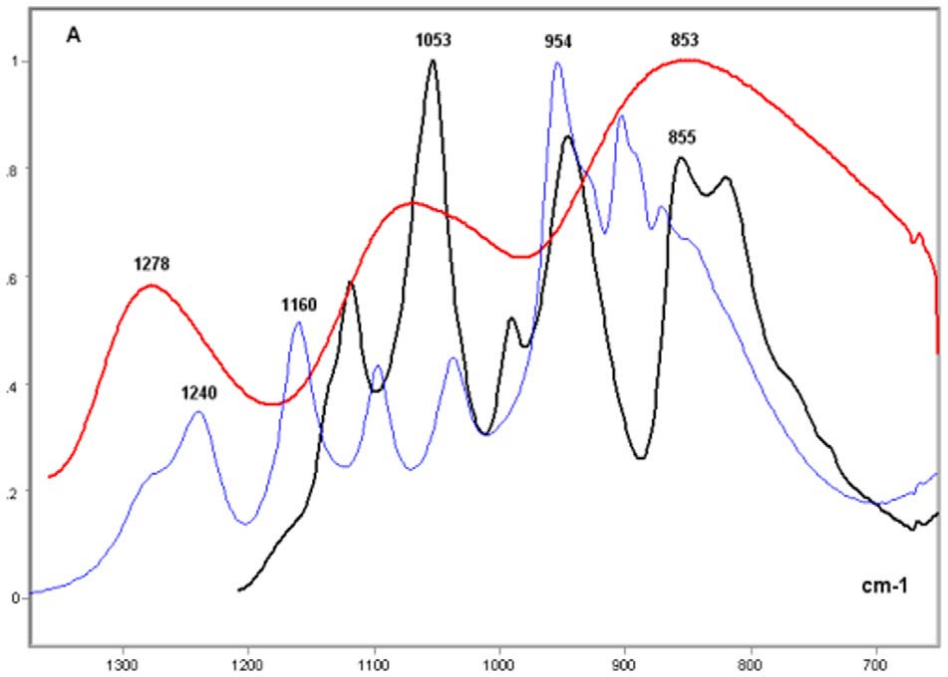
FT-IR spectral profiles of NaH_2_PO_4_, Na_2_HPO_4_, and KH_2_PO_4_. KH_2_PO_4_, red line; NaH_2_PO_4_, blue line; Na_2_HPO_4_, black line; X axis, wavenumbers in cm^−1^; Y axis, absorbances (A).

The NaH_2_PO_4_, Na_2_HPO_4_ and KH_2_PO_4_ FT-IR profiles were compared in the spectral range of ∼1300–700 cm^−1^ wavenumbers. The differences in the spectral positioning of the band peaks were found to vary as a function of the cationic components of these inorganic phosphates (Fig. 1). The spectral profile of KH_2_PO_4_ presented three smooth band peaks with increasing absorbance values from the highest to the smallest wavenumber regions (1278–1275 cm^−1^, ∼1074 cm^−1^ and ∼855 cm^−1^). These profile characteristics remained unchanged even if the profile was processed for peak fitting by the Grams software (data not shown). NaH_2_PO_4_ and Na_2_HPO_4_ exhibited several band peaks, with the highest positioned at ∼1053 cm^−1^ for Na_2_HPO_4_ and at 960–950 cm^−1^ for NaH_2_PO_4_. Conspicuous band peaks at 1240, 1160 and 1090 cm^−1^ were found only in the profile for NaH_2_PO_4_ (Fig. 1).

**Figure 2:**
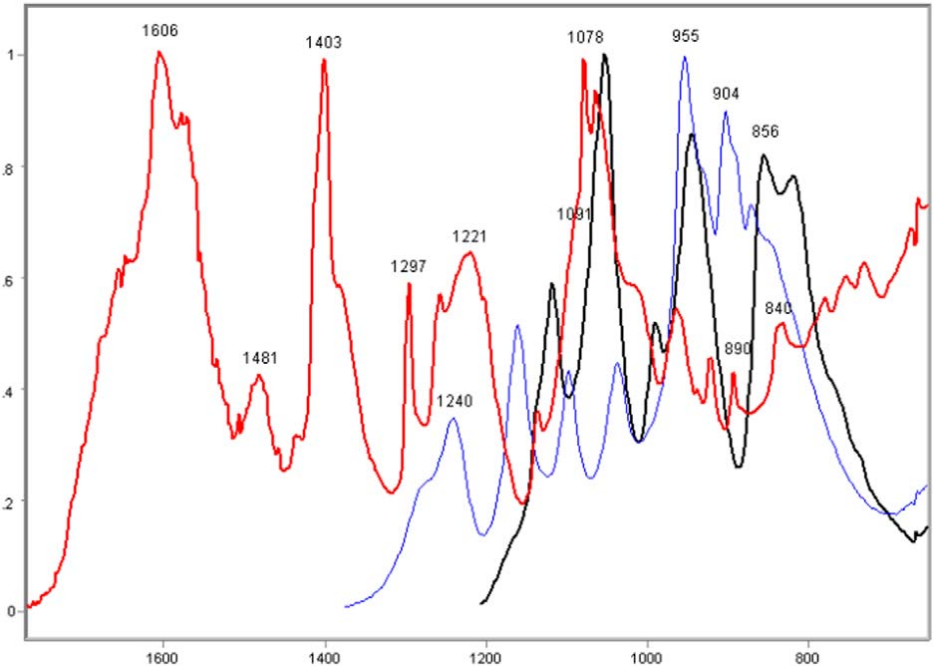
FT-IR spectral profile of calf thymus DNA compared to those of NaH_2_PO_4_, and Na_2_HPO_4_. DNA, red line; NaH_2_PO_4_, blue line; Na_2_HPO_4_, black line; X axis, wavenumbers in cm^−1^; Y axis, absorbances (A).

The comparison of the FT-IR spectral signature of the calf thymus DNA and sodium phosphates, revealed that although the band peaks at 1240 and 1090 cm^−1^ were not as sharp in the DNA as they were for NaH_2_PO_4_, the absorbances at these wavenumbers were relatively much more elevated in the DNA (Fig. 2). Absorption peaks in the ∼1606, 1480, 1400, 1300, 1220–1210, 1080, ∼1050, 890, and 840 cm^−1^ regions were evident in the FT-IR profile of the DNA; the peaks at ∼1600, ∼1400 and ∼1080 cm^−1^ were generally the highest (Fig. 2).

### Poly-L-K-, Poly-L-R- and Histone H1-Calf Thymus DNA Complexes

**Figure 3:**
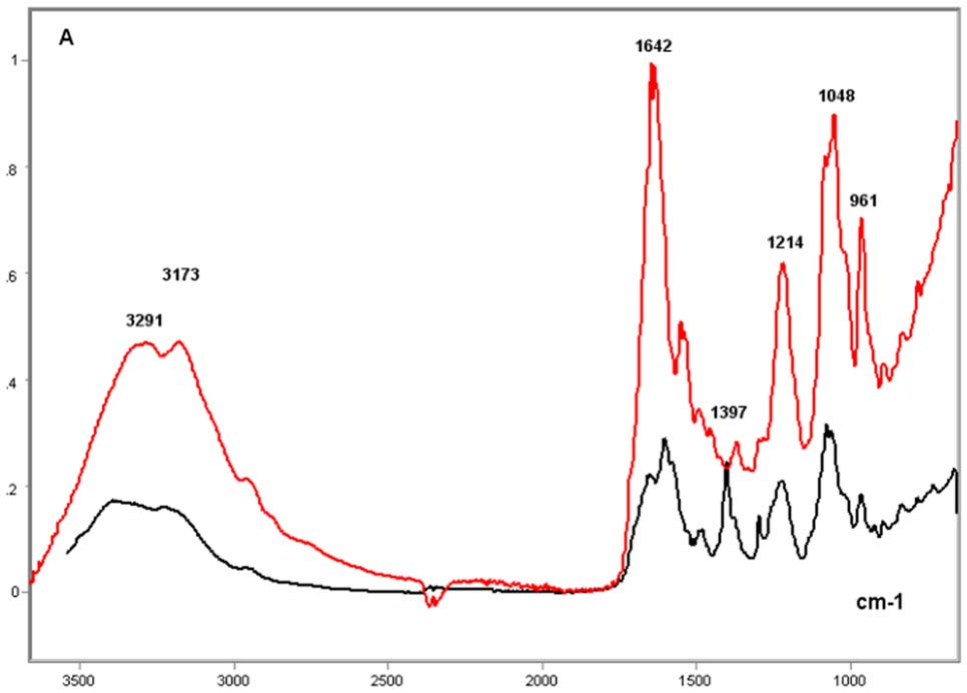
FT-IR spectral profile of poly-L-K-calf DNA complex compared to that of calf thymus DNA. Calf thymus DNA, black line; Poly-L-K-calf thymus DNA complex, red line; X axis, wavenumbers in cm^−1^; Y axis, absorbances (A).

In the complexes of DNA with poly-L-K and poly-L-R under the present experimental conditions, relatively higher absorption peaks and larger band areas were induced on the DNA IR spectral profile. The resulting FT-IR signature varied as a function of the polypeptide bound to the DNA (Figs. 3–6). The FT-IR profiles of poly-L-K, used here as a histone H1-mimicking polypeptide, and of the H1 histone were similar to each other in the 1700–1450 cm^−1^ wavenumber range and contained absorption peaks close to each other in the 1635–1615 cm^−1^ wavenumber range (data not shown).

**Figure 4 pone-0043169-g004:**
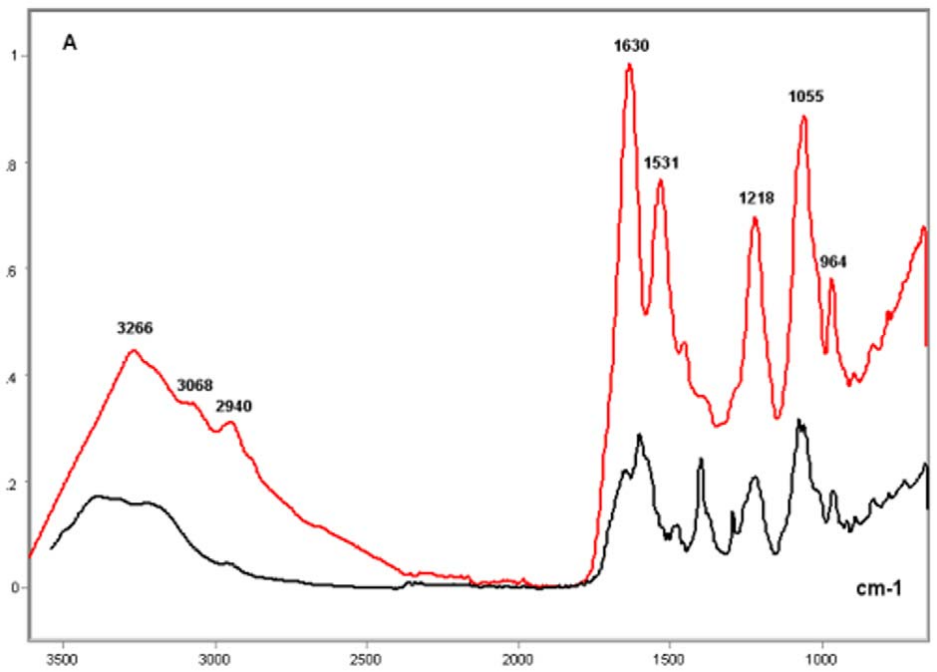
FT-IR spectral profile of histone H1-calf DNA complex compared to that of calf thymus DNA. Calf thymus DNA, black line; Histone H1-calf thymus DNA complex, red line; X axis, wavenumbers in cm^−1^; Y axis, absorbances (A).

**Figure 5 pone-0043169-g005:**
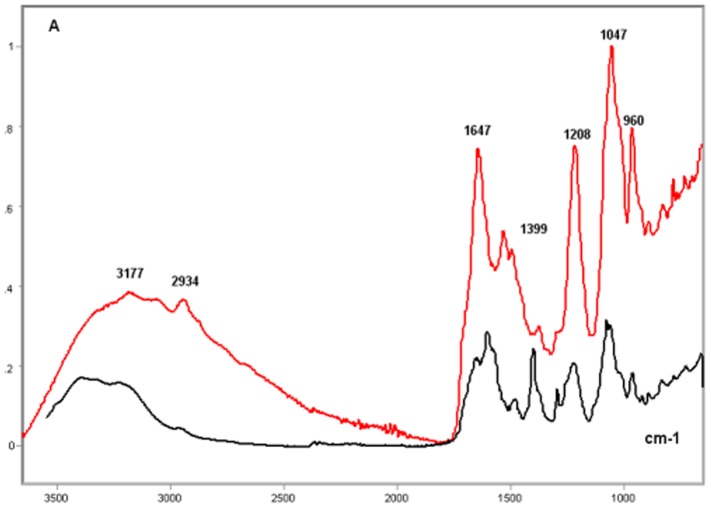
FT-IR spectral profile of the poly-L-R-calf DNA complex compared to that of calf DNA. Calf thymus DNA, black line; Poly-L-R-calf thymus DNA complex, red line; X axis, wavenumbers in cm^−1^; Y axis, absorbances (A).

**Figure 6:**
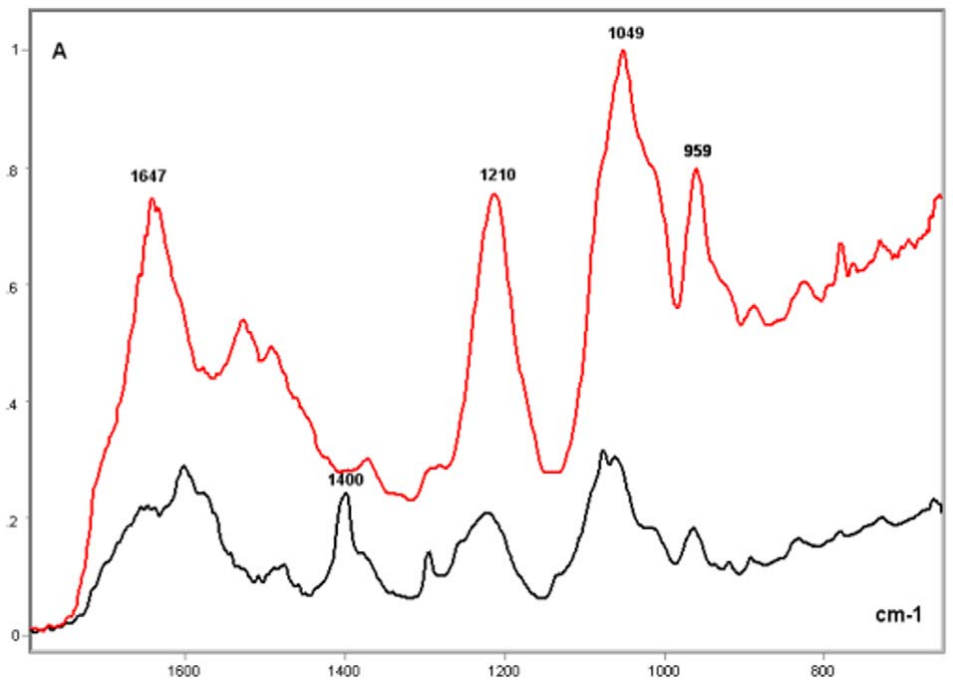
FT-IR spectral profile of the poly-L-R-calf DNA complex compared to that of calf thymus DNA. Detail of the IR spectral window in the 1800–700 cm^−1^ wavenumber range. Calf thymus DNA, black line; Poly-L-R-calf thymus DNA complex, red line; X axis, wavenumbers in cm^−1^; Y axis, absorbances (A).

The CH_2_-stretching vibration in the region 3000–2800 cm^−1^ of the IR spectra in the histone H1-DNA complex gained more intensity than in the poly-L-K-DNA complex, but less intensity than in the poly-L-R-DNA complex (Figs. 3–5). The vibrational intensity corresponding to the absorption peak positioned at 1530 cm^−1^ in the histone H1-DNA complex (Fig. 4) was more pronounced than that in the poly-L-K-DNA complex (Fig. 3). The band peak originally found at ∼1630 cm^−1^ in the poly-L-K and histone H1 spectra shifted to the 1645–1642 cm^−1^ region in the poly-L-K-DNA complex (Fig. 3), but remained positioned at the 1630 cm^−1^ region in the histone H1-DNA complex (Fig. 4).

The band peak at ∼1400 cm^−1^ for the DNA did not differ much from that in the poly-L-K-DNA complex (Fig. 3) but gained intensity in the histone H1-DNA and poly-L-R-DNA complexes (Figs. 4–6). Although the absorbances at 1225–1215 cm^−1^ were more pronounced after poly-L-K, histone H1 and poly-L-R were bound to the DNA, they were slightly higher in the poly-L-K-DNA and histone H1-DNA complexes (Figs. 3–6). The absorbances in the 1060–1050 cm^−1^ and ∼960 cm^−1^ wavenumber regions were higher for the poly-L-R-DNA complex compared with those for the poly-L-K-DNA and histone H1-DNA complexes, although the peaks in these regions were observed for all these complexes (Figs. 3, 5 and 6).

Numerous smaller peaks at wavenumbers <950 cm^−1^, including those at 890 and 840 cm^−1^, which occurred in the profile for pure DNA, gained intensity in the polypeptide-DNA complexes (Figs. 3–6).

When calculating the ratio obtained from the absorbance values evaluated at 1220 cm^−1^/1079 cm^−1^, which are closest to the wavenumbers used for the calculation of the v_as_/v_s_ PO_2_^−^ ratio [22], the values obtained for the polypeptide-DNA complexes were higher than those for the DNA alone (Table 3).

**Table 3:** The ν_as_ PO_2_^−^/ν_s_ PO_2_^−^ ratio for NaH_2_PO_4_, DNA, and DNA-polypeptide complexes.

Samples	ν_as_ PO_2_^−^/ν_s_ PO_2_^−^ ratio		Wavenumbers (cm^−1^) at which absorbances were obtained for the calculation of the ratio
	X	D	
Calf thymus DNA*	0.52		1225/∼1088
NaH_2_PO_4_	0.83		1240/1090
Calf thymus double-stranded DNA	0.67	0.02	1220/1079
Calf thymus single-stranded DNA	0.71	0.01	1221/1079
Calf thymus double-stranded DNA+poly-L-K	0.74	0.02	1220/1079
Calf thymus double-stranded DNA+histone H1	0.87	0.04	1220/1079
Calf thymus double-stranded DNA+poly-L-R	0.86	0.02	1220/1079
Salmon testis double-stranded DNA	0.54	0.01	1220/1079
Salmon testis double-stranded DNA+protamine	0.80	0.06	1220/1079

* [22]; SD, standard deviation; X, average; n, 10; ν_as_, antisymmetric stretching; ν_s_, symmetric stretching.

### Salmon Testis DNA and Protamine-DNA Complex

**Figure 7:**
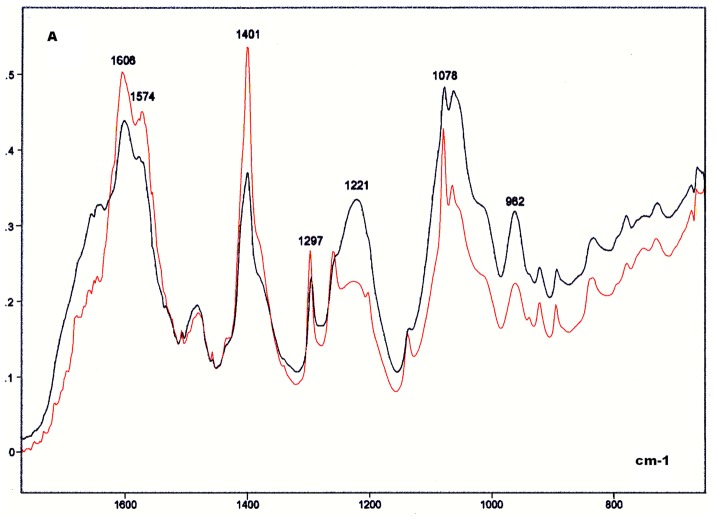
FT-IR spectral profile of the salmon DNA compared to that of the calf DNA. Detail of the IR spectral window in the 1800–700 cm^−1^ wavenumber range. Calf thymus DNA, black line; Salmon testis DNA, red line; X axis, wavenumbers in cm^−1^; Y axis, absorbances (A).

As with the calf thymus DNA, band peaks at ∼1606, 1480, 1400, 1300, 1080–1060, 960, 890, and 840 cm^−1^ were evident for the salmon testis DNA (Fig. 7). The peaks at ∼1608, 1574, 1400, and 1300 cm^−1^ were higher in the DNA from the salmon testis compared with those from the calf thymus. The peak at 1400 cm^−1^ was the highest in the IR spectral profile of the salmon testis DNA (Fig. 7). At ∼1220 cm^−1^, the absorption peak was more conspicuous for the DNA from the calf thymus. The absorbances at 1079–700 cm^−1^ were also higher for the DNA from the calf thymus compared with that from the salmon testis (Fig. 7). When calculating the ratio obtained from the absorbance values evaluated at 1220 cm^−1^/1079 cm^−1^, the value for the DNA from the salmon testis was lower than that from the calf thymus (Table 3).

**Figure 8:**
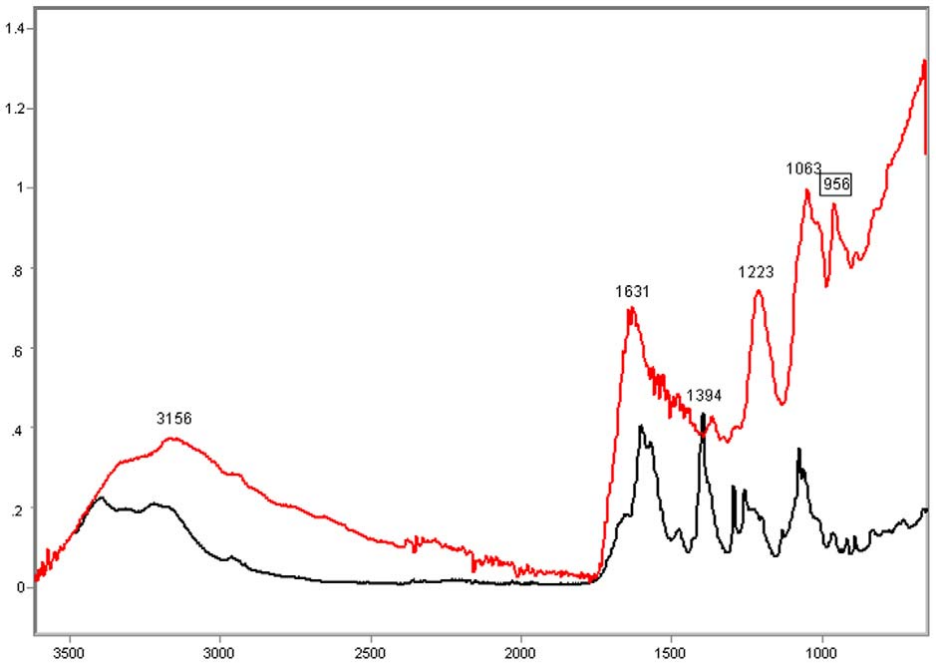
FT-IR spectral profile of the protamine-salmon DNA complex compared to that of the salmon DNA. Protamine-salmon testis DNA complex, red line; Salmon testis DNA, black line; X axis, wavenumbers in cm^−1^; Y axis, absorbances (A).

**Figure 9:**
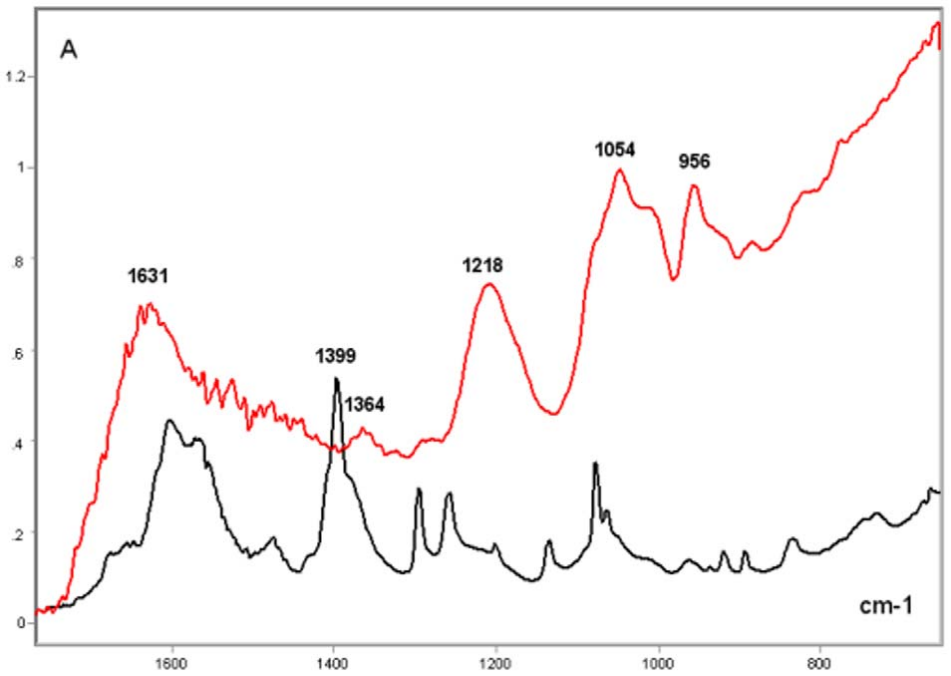
FT-IR spectral profile of the protamine-salmon DNA complex compared to that of salmon testis DNA. Detail of the IR spectral window in the 1800–700 cm^−1^ wavenumber range. Protamine-salmon testis DNA complex, red line; Salmon testis DNA, black line; X axis, wavenumbers in cm^−1^; Y axis, absorbances (A).

The FT-IR absorbances of the salmon testis DNA gained intensity, except at ∼1400 cm^−1^, after complexation with protamine (Figs. 8 and 9). The absorbance increase in the salmon DNA induced by protamine at wavenumbers <960 cm^−1^ was much higher than the absorbance increase induced by poly-L-R in the calf DNA (Figs. 5, 6, 8, 9). Protamine also increased the DNA absorbance ratio at the 1220 cm^−1^/1079 cm^−1^ wavenumbers (Table 3).

### Single- vs Double-Stranded Calf Thymus DNA

**Figure 10:**
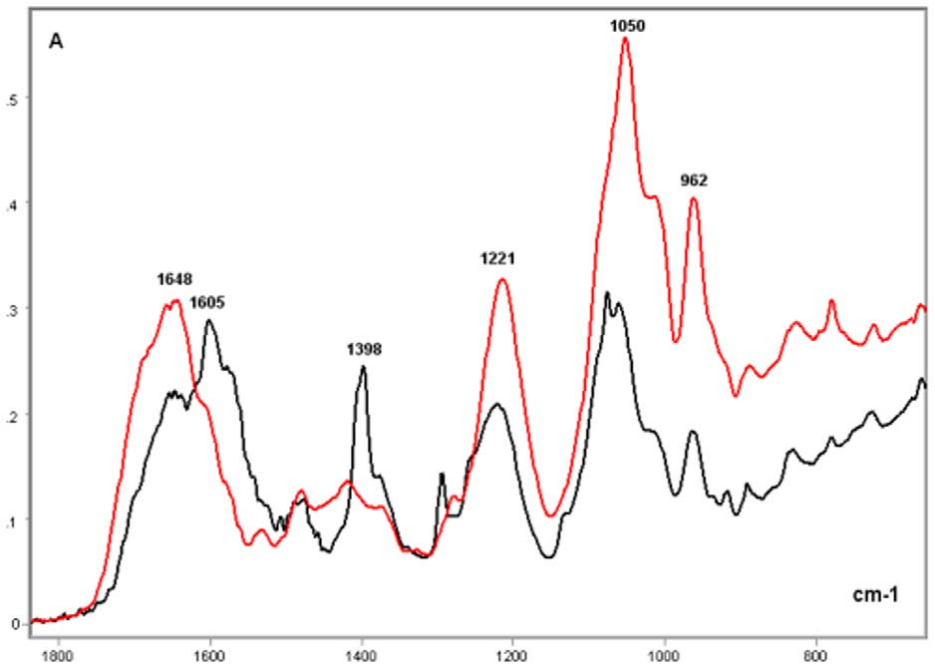
FT-IR spectral profiles of the double- and single-stranded calf thymus DNA. Double-stranded calf thymus DNA, black line; Single-stranded calf thymus DNA (red line); X axis, wavenumbers in cm^−1^; Y axis, absorbances (A).

**Figure 11:**
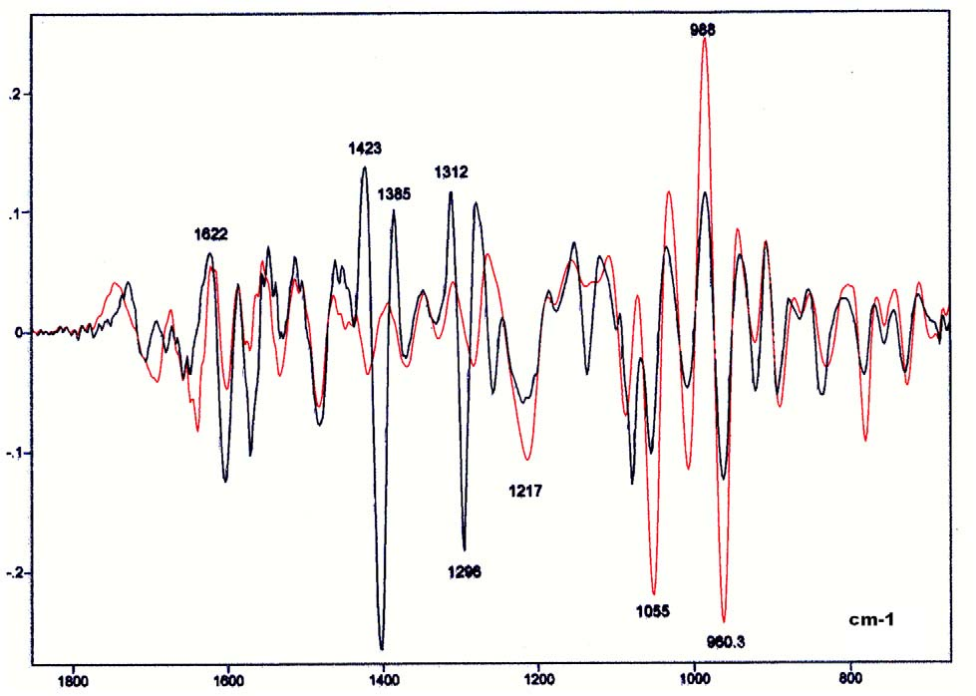
Second Savitzky-Golay derivative spectra of the profiles shown in **Fig. 10** for calf DNA. Double-stranded calf thymus DNA, black line; Single-stranded calf thymus DNA (red line); X axis, wavenumbers in cm^−1^; Y axis, second derivative.

The comparison of the spectral profiles of the single- and double-stranded thymus DNAs to each other revealed several differences (Figs. 10 and 11). In the single-stranded DNA, there was an apparent shift of the peak originally observed in double-stranded DNA at ∼1600 cm^−1^ to the left-side of the spectrum (∼1650 cm^−1^), a maintenance of the band peak intensity at 1480 cm^−1^, and a deep reduction in the intensity at ∼1400 cm^−1^ (Fig. 10). A reduction in the intensity at ∼1300 cm^−1^ was also observed. Additionally, higher absorbances, but no peak shift, in the 1200–800 cm^−1^ wavenumber range were evident in the single-stranded DNA. These observations were validated in the averaged second derivative spectra (Fig. 11).

The ratio for the absorbance values obtained at 1220 cm^−1^/1079 cm^−1^ in the single-stranded DNA was higher than that found for the double-stranded DNA (Table 3).

In all of the cases reported above, the absorbances corresponding to ν_as_ PO_2_^−^ (∼1240–1220 cm^−1^) were lower than those corresponding to v_s_ PO_2_^−^ (∼1090–1079 cm^−1^) (Table 3).

## Discussion

The band peaks observed at 1240 and 1090 cm^−1^ in the profile of the NaH_2_PO_4_ coincide with or are close to those proposed for the DNA antisymmetric (ν_as_) and symmetric (ν_s_) PO_2_^−^ stretching, respectively [5], [13], [18], [20], [23]–[25]. Interestingly, sharp peaks at these wavenumbers in the DNA samples investigated here were not as evident as those in NaH_2_PO_4_ (or they were shifted to smaller frequencies) despite the absorbances being higher in the DNA samples. This effect may be promoted by a more complex vibrational influence of the molecular microenvironment on the DNA components under the experimental conditions. The peak at 960–954 cm^−1^ found in the DNA samples, especially those from the salmon testis, was also evident in NaH_2_PO_4_ and may be assigned to O-P-O bending [13]. These spectral characteristics were not found in the other inorganic phosphates analyzed here, thus supporting the idea that different cations differently affect the vibrational characteristics of the phosphate anions in inorganic phosphates.

The vibrational intensity corresponding to the absorption peak at ∼1220 cm^−1^, which is representative of the antisymmetric stretching of the PO_2_^−^ groups in the DNA, is higher in the calf DNA than in the salmon DNA. The same result is assumed to occur regarding the symmetric stretching of the PO_2_^−^ groups, provided that the absorption peak at ∼1080–1078 cm^−1^ is representative of this vibrational characteristic. The presence of absorption bands in the ∼1220 and 1080 cm^−1^ regions indicates the secondary conformation in the *B*-form for both DNA types [24].

Differences in the base composition between calf and salmon DNA samples have been reported. While the DNA of the calf thymus has a plurimodal base composition, that of the salmon testis has an AT-biased (∼60%) composition [26], [27]. Considering the association of a peak at 1400 cm^−1^ with all DNA bases [22], the vibrational intensity at ∼1400 cm^−1^, which in the salmon DNA is more elevated than it is in the calf DNA, could be caused by the AT-richness. This idea is reinforced by the observation that the band peaks at 1608 and 1574 cm^−1^, which were also more pronounced in the salmon testis DNA, are assigned to adenine [22], [25], [28]. Assuming that the peaks that were slightly shifted from these wavenumbers in the calf thymus DNA are also attributed to adenine, there is an increase in the vibrational frequency associated with this base under single-stranded conditions. However, the vibrational intensity associated with the absorbance peak at 1400 cm^−1^, assigned to all DNA bases, showed a decrease as substantial as that for the vibrational intensity at ∼1300 cm^−1^, which has been attributed to cytosine [25].

In the 1250–800 cm^−1^ wavenumber region, there was generally no change in the vibrational frequency, but a deep increase in the vibrational intensities did occur in the single-stranded calf DNA, including those peaks corresponding to the PO_2_^−^ antisymmetric stretching (1220 cm^−1^), O-P-O bending (960 cm^−1^) [13], deoxyribose ring vibration (900 cm^−1^) [23] and main S-type sugar marker (840 cm^−1^) [23], [29]. A peak associated with PO_2_^−^ symmetric stretching was not precisely positioned at 1090–1085 cm^−1^ in the single-stranded DNA; however, the absorbances at these wavenumbers gained intensity, and a peak was observed at ∼1050 cm^−1^. Although the antisymmetric PO_2_^−^ stretching band is a characteristic marker of the DNA backbone conformation, with the band peak at ∼1220 cm^−1^ associated with the *B-*form double helix, the vibrational properties associated with the nucleic acid pentose, as reported in the case of RNA [30], may contribute to the absorbances at this wavenumber in the single-stranded DNA.

The peak at 1480 cm^−1^ identified in the DNAs from calf thymus and salmon testis is assigned mainly to purine imidazole ring vibrations [19], [23].

Under the present experimental conditions, the vibrational characteristics of the DNA PO_2_^−^ groups were differently affected by the different polypeptides bearing cationic groups available for DNA binding (histone H1, protamine and polypeptides mimicking lysine-rich and arginine-rich histones). On average their absorbances and band areas increased. The poly-L-K and poly-L-R affected the structure of the calf thymus DNA differently because their binding to the nucleic acid molecule differs, as previously demonstrated by other methodologies, including critical electrolyte concentration studies, in *in vitro* models [31] and in cell chromatin *in situ* [32].

The perturbations introduced by the poly-L-K and poly-L-R in the calf thymus DNA vibrational characteristics in the wavenumber region less than 1633 cm^−1^, which is contained in the IR fingerprint region [33], are most likely caused by the specific compositions of the chain residues on these polypeptides and their intermolecular influences inside each of the DNA-polypeptide complexes. These perturbations are intensified in the FT-IR fingerprint, especially at wavenumbers <960 cm^−1^, as in the case of the protamine-salmon DNA complex.

Protamine is a small arginine-rich protein that forms a complex with DNA in the sperm cells of many animal species, notably fish such as salmon [34]. The enhancement of the vibrational characteristics in the IR fingerprint for the poly-L-R-calf thymus DNA complex or for the protamine-salmon DNA complex is assumed to be promoted by the ordered arrangement of the polypeptide molecules when they are complexed with DNA. This arrangement is even more effective than the arrangement in the poly-L-K-DNA complex because poly-L-R and protamine are known to better optimize DNA conformations [35]–[37]. Although previous data on the dispersion of birefringence and linear dichroism have demonstrated that poly-L-K and poly-L-R bind DNA in a specific orientation, the optical anisotropy properties of the DNA become much more intense after protamine binding [35], [38]. Protamination of the DNA is thought to facilitate the higher-order packaging of the DNA in sperm cells, giving rise to toroidal chromatin structures [36], [39]. The number of clustered arginine residues in the DNA binding domain of the protamine is a primary factor affecting the condensation and stability of the DNA-protamine complex [40]. In the case of the poly-L-R- and protamine-DNA complexes, no apparent effects were induced on the frequency of the antisymmetric stretching vibration of the DNA PO_2_^−^, but the intensity of this vibration became very high. This result was most likely due to an enhancement of the molecular order of the complexes.

Our results showed that the DNA band peak at 1224 cm^−1^, which is assumed to be associated with PO_2_^−^ ν_as_ and is a marker of the *B*-form of DNA, was sensitive to the backbone conformation (at least in solution) [23], [24] and moved slightly to the right-side of the IR spectrum (1218 cm^−1^) after poly-L-K binding. This finding indicates that the poly-L-K binding induces a shift in the vibrational frequency of the DNA PO_2_^−^ groups to lower values, which is in agreement with a previous report [24]. In addition, there was an indication that the poly-L-K induces a significant increase in the intensity of this vibration because the absorbances at the corresponding wavenumber region increased in the poly-L-K-DNA complex. The findings for the poly-L-K, poly-L-R, and protamine-DNA are in agreement with those in the report by Cherng [24] who showed that the band at 1224 cm^−1^ - corresponding to PO_2_^−^ ν_as_, changed after DNA complexation with polymers; it exhibits a greater change in either the band position or intensity after DNA condensation. A major increase in the intensity and shifting of the PO_2_^−^ antisymmetric band in DNA spectra has also been reported upon cationic lipid complexation [28]. The intensity of the antisymmetric stretching of the PO_2_^−^ groups was always higher than that of the symmetric stretching of these groups under all the experimental conditions used in the present investigation.

The absorbance increase at 1645–1636 cm^−1^ in the FT-IR spectral profile of the calf DNA induced after poly-L-K or histone H1 binding, which was greater than that induced after poly-L-R binding or in the salmon testis DNA after protamine binding, is suggestive of the effects due to the vibrational stretching of the polypeptide amide I; this increase results from its richness in lysine residues [5], [18], [41], [42]. Peaks corresponding to the vibrational properties of the amide II and III components [5], [18], [43] were not conspicuous in the polypeptide-DNA complexes, although increased absorbances at ∼1560 and 1245 cm^−1^ were evident in these IR spectral regions. At the ∼3000 cm^−1^ wavenumber, where the vibration of the polypeptide CH_3_ or CH_2_ groups would be expected [13], [18], a peak that shifted to a lower frequency was verified in the poly-L-K- and histone H1-DNA complexes.

## Conclusions

1. The FT-IR spectral profiles obtained with an IR microspectroscope equipped with an attenuated total reflection diamond objective are an important tool for establishing the vibrational characteristics of the individualized components of chromatin, such as DNA and DNA-polypeptide complexes, in dried samples.
2. Histone H1, protamine and histone-mimicking macromolecules differently affect the vibrational characteristics of the chemical groups (especially, PO_2_^−^) in the DNA, thereby generating different FT-IR band positions or intensities. The shift of the DNA PO_2_^−^ ν_as_ to a lower frequency (1218 cm^−1^) in association with an increased intensity of this vibration is particularly affected by the lysine-rich histones. The richness of the lysine residues in these polypeptides also contributes to the increase in the vibrational stretching relative to the amide I group identified at 1645–1636 cm^−1^.
3. Even in simple molecules such as inorganic phosphates, the vibrational characteristics of the phosphate anions are differently affected by different cations, as assessed by the FT-IR analysis.
4. There is an enhancement of the vibrational characteristics of the FT-IR fingerprint, especially at wavenumbers <960 cm^−1^, in the complexes of DNA with arginine-rich polypeptides, including protamine, most likely because of an optimization of the DNA conformation promoted by the ordered arrangement of these polypeptides.
5. Although DNA with different base compositions can generate different FT-IR signatures, the vibrational intensities relative to the specific nucleobase groups are masked in the profiles provided by the polypeptide-DNA complexes. In isolated chromatin or cell nuclei, differences related to the specific base compositions of the DNA may not be identified by band peaks corresponding to the vibrational frequencies or intensities of these groups because they may not be perceived.
6. Although the intensity of the antisymmetric stretching of the DNA PO_2_^−^ -groups was always greater than that of the symmetric stretching of these groups, the ν_as_ PO_2_^−^/ν_s_ PO_2_^−^ ratio and the absorbance values corresponding to ν_s_ PO_2_^−^ indicate an increase in the ν_as_ in the DNA after its complexation with histone H1, histone-like polypeptides or protamine. In isolated DNA, the ν_as_ PO_2_^−^/ν_s_ PO_2_^−^ ratio may be able to discriminate among different DNA compositions and the DNA single-stranded form.
7. The FT-IR profile of the isolated single- and double-stranded DNA samples can be discriminated from each other. However, it may be anticipated that in chromatin or cell nuclei subjected to denaturation procedures, the generation of single-stranded fragments caused by the opening of the double helix may not be distinguishable because the markers usually associated with the DNA *B*-form may not appear to be affected, possibly because of the vibrational contribution of other DNA components (pentoses?).
8. The present results are intended to be a contribution to support the forthcoming understanding and interpretation of the differences in the FT-IR signatures for chromatin and whole cell nuclei. FT-IR studies on more complex aggregates of chromatin elements (methylated DNA, modified histones, sirtuins and transcription factors) will certainly bring additional knowledge to this field.

## Materials and Methods

### Materials

Sample preparations from NaH_2_PO_4_, Na_2_HPO_4_, KH_2_PO_4_, calf thymus double- and single-stranded DNA, salmon testis DNA, poly-L-lysine hydrochloride (poly-L-K) (mol wt 30,000), poly-L-arginine hydrochloride (poly-L-R) (mol wt 15,000), protamine sulfate (all purchased from Sigma-Aldrich, St. Louis, USA), and calf thymus histone H1 (Calbiochem, San Diego, USA) were used. Among the possible DNA types differing in nucleobase composition, polymodal nucleobase-containing calf thymus DNA and salmon testis AT-biased DNA were selected as standard materials for the present study because of their ease of being purchased. In addition, calf DNA is a good representative of mammalian nucleic acid, and salmon DNA most naturally binds salmon protamine.

NaH_2_PO_4_, Na_2_HPO_4_, and KH_2_PO_4_ powders were spread on glass slides for examination. The double- and single-stranded DNA samples were prepared similarly as follows. A hyper-polymerized calf thymus DNA solution was obtained by dissolving 6 mg of DNA in 1 mL of 0.9% NaCl solution, and the preparation was left in the refrigerator until complete DNA dissolution, which corresponded to the formation of a liquid crystal fibro-colloid, was obtained. This material was spread on the surface of the glass slides and left in the refrigerator for preliminary drying. Then, it was removed from the refrigerator, air dried at 37°C, rinsed in 80% ethanol to remove the NaCl crystals, and air dried again at 37°C. The characteristic optical anisotropy of the DNA (negative birefringence) was identified on the fibro-colloidal liquid crystal and on the material spread on the glass slides using an Olympus BX-51-PBX2 polarization microscope equipped with Image Pro-Plus 6.3 software before examination with the IR microspectroscope.

Complexes of the calf thymus DNA with poly-L-K, poly-L-R and histone H1- and complexes of the salmon testis DNA with protamine sulfate were also examined. In all these cases except the histone H1-DNA complex, one drop of a DNA solution prepared in 0.9% NaCl as mentioned above, and one drop of a 0.5% aqueous solution of each of the mentioned polypeptides/proteins were mixed, forming filaments that were spread with a needle on glass slides. The samples were then preliminarily dried in the refrigerator, air dried at 37°C, rinsed in 80% ethanol, and dried again at 37°C.

In the case of the histone H1-DNA complex, solutions of 5 mg of calf thymus histone H1 dissolved in 200 µL of milliQ water and of 4.4 mg of calf thymus hyperpolymerized DNA dissolved in 1.3 mL of 0.9% NaCl were left in the refrigerator overnight until complete dissolution. Next, the H1 solution was poured over the DNA solution, giving rise to a whitish filamentous precipitate that was left again in the refrigerator overnight. This precipitate was spread as filaments over slides and kept in the refrigerator until it was nearly dry, and then it was removed from the refrigerator, air dried at 37°C, rinsed in 80% ethanol to remove the NaCl crystals, and dried again at 37°C.

### FT-IR

The FT-IR spectral acquisition of the dried sample preparations on glass slides was performed using the Illuminat IR II™ microspectrometer (Smiths Detection, Danbury, USA) equipped with a liquid-cooled mercury-cadmium-telluride (MCT) detector and Grams/AI 8.0 spectroscopy software (Thermo Electron Co., Waltham, USA). An attenuated total reflection diamond objective (ATR, magnification 36×) was employed. The performance validation of the equipment used a low signal-to-noise ratio (7929∶1) [41].

The measurement site area was 50 µm per side; the absorbances of the samples and background were measured using 64 scans for each preparation. The spectral absorption signatures of the preparations at wavenumbers between 4000 cm^−1^ and 650 cm^−1^ were obtained with a spectral resolution (a measure of how well the closely spaced spectral features were distinguished) of 4 cm^−1^. Ten spectral profiles were obtained for each sample. Each spectral profile was subjected to baseline correction and normalized with respect to its highest peak with the Grams software. An average profile was then obtained for each sample using the Grams software. For the comparative analysis of the FT-IR spectral profiles of the double- and single-stranded DNA, and to obtain more detailed absorbance information at specific wavenumbers as in the reports by Bird et al. [44] and Zelig et al. [18], Savitzky-Golay's second derivative spectra were also determined.
